# Comparison of the Short and Long-Term Outcomes of Endovascular Repair and Open Surgical Repair in the Treatment of Unruptured Abdominal Aortic Aneurysms: Meta-Analysis and Systematic Review

**DOI:** 10.7759/cureus.9683

**Published:** 2020-08-12

**Authors:** Othman AlOthman, Suleiman Bobat

**Affiliations:** 1 Surgery, School of Medicine, University of Nottingham, Nottingham, GBR; 2 Vascular Surgery, Queen’s Medical Centre, Nottingham, GBR

**Keywords:** endovascular repair, evar, open surgical repair, osr, abdominal aortic aneurysm, aaa, vascular surgery

## Abstract

Background

Although the initial results of endovascular repair (EVAR) were promising, a comparison of its long-term efficacy against open surgical repair (OSR) remains largely elusive, and late-onset adverse events have not been systematically evaluated. Since OSR and EVAR are currently the only treatment options available in the management of abdominal aortic aneurysms (AAAs), the main question arising in clinical practice is whether EVAR or OSR confers more favourable short and long-term outcomes for patients presenting with unruptured AAAs.

Aims

The present meta-analysis aims to draw a head-to-head comparison between EVAR and OSR and facilitate the formulation of an evidence-based approach to the clinical management of unruptured AAAs.

Methods

A systematic review was conducted using three databases to identify all relevant studies with comparative data on EVAR vs. OSR. All-cause mortality was the primary outcome. Procedural outcomes, such as stroke, myocardial infarction, renal complications, rupture, and reintervention rates, were determined as secondary outcomes.

Results

Sixteen studies were included for comparative analysis, including four randomised-controlled trials and six non-randomised comparative clinical trials. EVAR conferred a clear perioperative survival advantage as compared to OSR (P < 0.00001). However, this survival advantage did not persist beyond two years post-procedure; all-cause mortality rates were comparable between the two treatment groups at two years (P = 0.09), four years (P = 0.58), and six years (P = 0.88) post-procedure. Although no statistically significant differences in aneurysm-related mortality, postoperative stroke, or myocardial infarction were identified, the OSR group had a statistically significant higher rate of postoperative renal complications. On the other hand, there was a statistically significant higher rate of rupture and reintervention following EVAR.

Conclusion

Whether the initial survival advantage afforded by EVAR is sufficient to justify the long-term risk of rupture, reintervention, and long-term mortality should be determined on a case-by-case basis by the multidisciplinary team overseeing the clinical care of the patient. Currently, it is reasonable to conclude that EVAR is as efficacious as OSR, but it would be invalid to claim it as superior. Ultimately, longer follow-up data must be presented before any definitive conclusions can be established for this potentially revolutionary technique. Presently, one can neither advocate nor refute EVAR over OSR.

## Introduction

An abdominal aortic aneurysm (AAA) is clinically defined as an abnormal, permanent, balloon-like dilation of the abdominal aorta that is 50% greater than its normal diameter [1]. In England and Wales alone, AAAs account for 4,000 annual deaths, rendering it the 10th leading cause of death in men aged over 55 [2]. Furthermore, AAA is among the most expensive cardiovascular diseases (CVDs) to treat; it imposes an economic burden of £140 million and significantly reduces patients’ life expectancy [3]. With the accumulating epidemiological evidence suggesting the continuation of AAAs to dominate morbidity trends, the prevalence of the disease is only set to steadily increase in the foreseeable future. The resultant increasing prevalence, patient hospitalisation, and economic consequences may thus contribute to a significant, yet perhaps underappreciated, burden on public health.

Historically, the limited success of elective AAA repairs directly translated into an overwhelming risk of death [4]. However, following recent breakthroughs in medical advances, endovascular repair (EVAR) and open surgical repair (OSR) have shown considerable potential in reducing operative risks and enhancing long-term outcomes. Currently, OSR and EVAR stand as the only two treatment methods available in the management of AAAs. The main question arising in clinical practice is whether OSR or EVAR confers more favourable short and long-term outcomes for patients with unruptured AAAs.

Although the initial results of EVAR were promising, a comparison of its long-term efficacy against OSR remains largely elusive and late-onset adverse events have not been systematically evaluated. Consequently, the present meta-analysis aims to explore, analyse, and compare the application of EVAR against OSR in the management of unruptured AAA. It is hoped that this project will enhance the clinical utility of OSR and EVAR by identifying trends in favour of either treatment method, thereby facilitating the formulation of an evidence-based approach to the clinical management of AAAs.

## Materials and methods

Literature search

A comprehensive literature search was performed in the PubMed and Embase databases. The search terms used included: “endovascular repair AND open repair AND abdominal aortic aneurysms”, “endovascular AND open surgical repair”, “management of unruptured abdominal aortic aneurysms”, and “outcomes following abdominal aortic aneurysm repair”. The search was limited to articles published in English. Terms related to the outcomes of interest (e.g. all-cause mortality, postoperative complications, etc.) were not used as search terms to prevent any restrictions on database search results. The search was refined using the PICO framework for clinical questions:

- Population: patients with an AAA who were eligible for surgical intervention. - Intervention: the main intervention could be taken to be EVAR. - Comparison: given the above, the comparison group would then be the OSR cohort. - Outcome: outcomes intended for comparative analysis are covered later in this section. Since EVAR is a relatively novel surgical technique, no date limits or other filters were applied.

Titles and abstracts were screened and then either excluded or included for full-text review, depending on their relevance to the scope of this review. In addition, a manual search of the reference list of the relevant literature was performed to identify any additional papers of interest, and duplicates were removed using EndNote.

Inclusion and exclusion criteria 

Inclusion criteria set were that the study must have compared outcomes following EVAR and OSR in patients presenting with unruptured AAAs; have a mean follow-up period of at least 2 years; be a randomised controlled trial (RCT) or a non-randomised comparative study; and report outcomes >30 days post-procedure. Given that clinical trials with small sample sizes may be statistically inconclusive, a minimum number of 200 and 500 patients was required for RCTs and non-randomised trials, respectively. Studies were excluded from the review if they were case studies, conference presentations, systematic reviews, expert opinions, animal models, or if they did not specifically compare the outcomes following OSR and EVAR in the management of unruptured AAAs. Furthermore, because a short follow-up period would limit drawing any conclusion regarding the long-term durability of both treatments, studies with a mean follow-up period of less than two years were omitted.

Study selection

**Figure 1:**
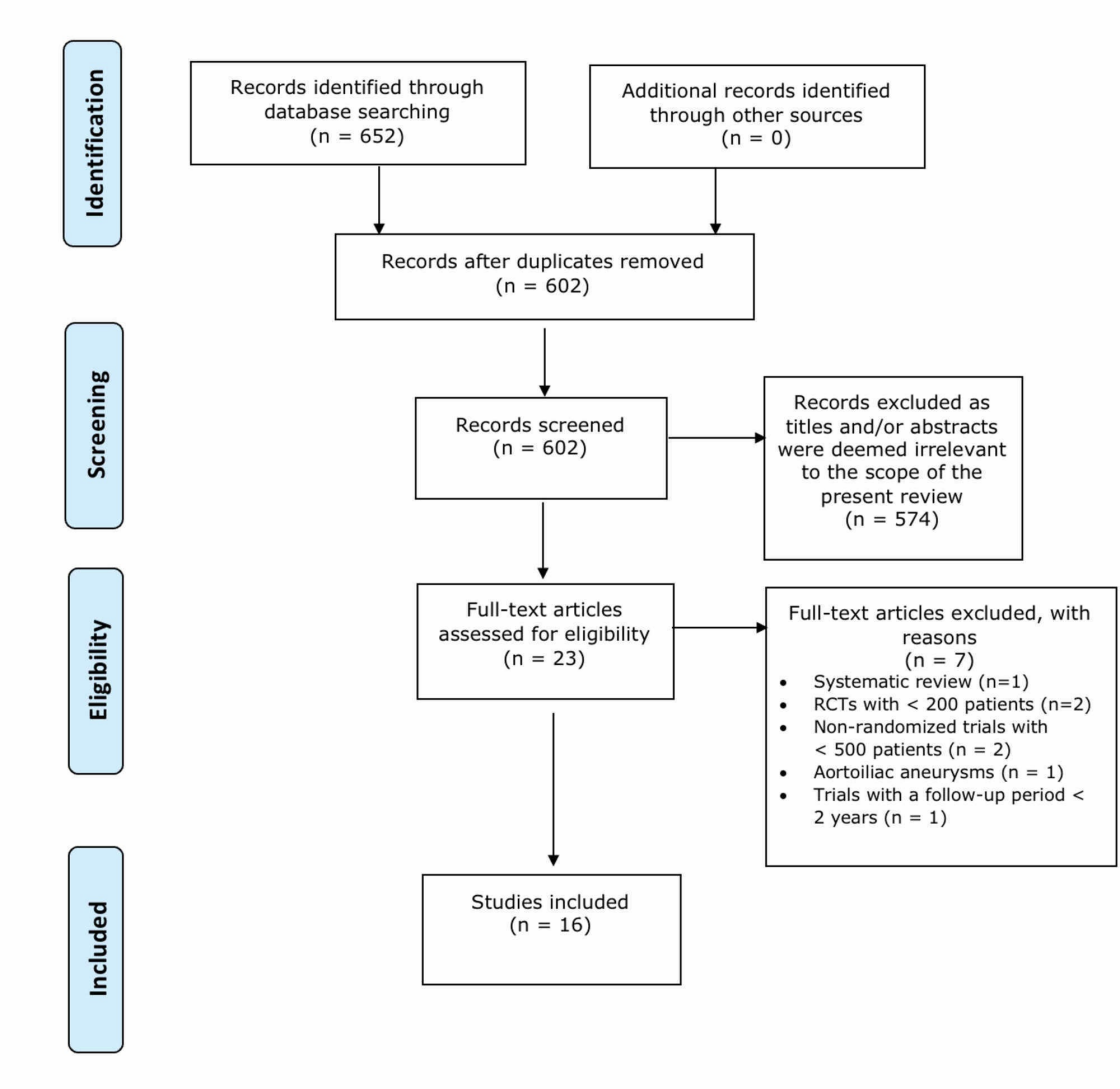
PRISMA 2009 flow diagram for the literature search and identification of relevant studies

The literature search retrieved 652 papers (Figure 1). Following the removal of duplicates, titles and abstracts were screened and either excluded or included for full-text analysis. Full-text analysis of 23 papers was performed. Of the 23 papers, seven were excluded for the following reasons: one was an RCT with <200 patients, three were non-randomised trials with fewer than 500 patients, one non-randomised trial had a follow-up trial of less than two years, one clinical trial investigated aortoiliac aneurysms, and one was a systematic review. Sixteen papers were retained after applying the inclusion and exclusion criteria and were, therefore, included for further comparative analysis. Of the studies included, 10 studies reported outcomes from four independent clinical trials at different time intervals, in addition to six other clinical trials [5-20] (Table 1).

**Table 1:** Studies included in the present meta-analysis OSR: open surgical repair; EVAR: endovascular repair; RCT: randomised controlled trial; ACE: Acarbose Cardiovascular Evaluation; VSS: Paracor Ventricular Support System; OVER: Open versus Endovascular Repair; EVAR1: EVAR trial 1

Trial	Study Design	Total Number of Patients	OSR Patients	EVAR Patients	Single or Multicentre	Study Quality Score
ACE [5]	RCT	299	149	150	Multicentre	Appendix
Behrendt et al. [6]	Non-randomized	4950	1457	3493	Multicentre	8/9
DREAM [7-9]	RCT	351	178	173	Multicentre	Appendix
EVAR1 [10-13]	RCT	1252	626	626	Multicentre	Appendix
Medicare [14]	Non-randomized	45,660	22,830	22,830	Multicentre	8/9
OVER [15-16]	RCT	881	437	444	Multicentre	Appendix
Peripheral Vascular Surgery Society (PVSS) [17]	Non-randomized	677	417	260	Single	7/9
Southern Association for Vascular Surgery (SAVS) [18]	Non-randomized	1986	920	1066	Single	7/9
Swedish Vascular Registry Trial (SwedVasc) [19]	Non-randomized	3777	2922	855	Multicentre	8/9
Vascular Study Group of New England (VSGNE) [20]	Non-randomized	1546	476	1070	Multicentre	8/9

Outcomes of interest 

Outcome measures of interest, following EVAR and OSR in patients with unruptured AAAs, intended for comparative analysis across the relevant literature include all-cause mortality, aneurysm-related mortality, rate of reintervention, selected postoperative complications (i.e. stroke, myocardial infarction, and renal complications), and rates of rupture when applicable. Separate analyses were performed for all outcomes of interest, with the inclusion of all clinical trials that had reported data on the outcome under analysis.

Statistical analysis

Data were extracted from each paper and then analysed using Review Manager 5.3 (The Nordic Cochrane Centre, The Cochrane Collaboration, 2014, Copenhagen) [21]. For the purpose of the meta-analysis, outcomes reported as a percentage were converted to raw numbers. Outcomes that are dichotomous in nature are reported as odds ratio (OR) with a 95% confidence interval (CI). For continuous data, mean differences with standard deviation (SD) are presented. A p-value of ≤ 0.05 was set as the threshold to determine the statistical significance of data. Since 10 of the included papers reported outcomes from four clinical trials at different time intervals, multiple publications relating to the same outcome from a single clinical trial were common. However, when identified, outcome data from the most recently published paper were included. With RCTs, an analysis was performed on an intention-to-treat basis; patients were analysed according to their original treatment group assignment.

The chi-square test is the statistical test used to assess the degree of association between the treatment method and all outcomes of interest. The Mantel-Haenszel method and a random- effect model were applied, owing to inherent variability between the baseline characteristics of the study populations in each paper. The heterogeneity among the different clinical trials was assessed using the I2 statistics. This represents the percentage of the total variation of the treatment effect across the studies that is not attributable to chance or random error. The value of I2 lies between 0% and 100%, with larger values reflecting increasing heterogeneity due to real differences in the population in question, interventions, or outcomes [22].

Risk of bias assessment 

As recommended by the literature, a study tool assessment was used to assess the quality of the studies included in the present review. The quality of non-randomised comparative studies was evaluated using the 9-Point Newcastle-Ottawa Scale, which assesses the representativeness of the sample population, the methods of patient selection, the comparability between the treatment groups, and the methods of outcome assessment. The Cochrane Collaboration’s tool was used to assess the risk of bias in RCTs. Seven potential sources of bias were assessed and assigned a high, low, or unclear risk of bias accordingly: random sequence generation (selection bias), concealment of allocation (selection bias), blinding of outcome assessment (detection bias), blinding of the participants and personnel (performance bias), incomplete outcome data (attrition bias), selective outcome reporting (reporting bias), and any other potential source of bias (e.g. industrial bias) (Table 2).

**Table 2:** Assessment of the quality of randomised controlled trials included NA: Given the impracticality of attempting treatment blinding in such surgical procedures, blinding of participants and personnel was not applicable. All RCTs had an unclear risk of bias under “other sources of bias”. This is mainly due to conflicts of interest. OSR: open surgical repair; EVAR: endovascular repair; RCT: randomised controlled trial

	Random Sequence Generation	Allocation Concealment	Blinding of Outcome Assessment	Blinding of Participants and Personnel	Incomplete Outcome Data	Selective Outcome Reporting	Other Sources of Bias
ACE	low	low	moderate	NA	low	low	unclear
EVAR1	low	low	moderate	NA	low	low	unclear
DREAM	low	low	low	NA	low	low	unclear
OVER	low	low	low	NA	low	low	unclear

## Results

The database search revealed 652 studies of which 16 were retained after the initial removal of duplicates and applying the inclusion and exclusion criteria [5-20]. Of the studies included, 10 RCTs reported outcomes from four clinical trials at different time intervals, in addition to six other non-randomised trials.

A total of 61,379 patients, with a mean age of 74, were analysed in the present meta-analysis: 30,412 receiving OSR and 30,967 receiving EVAR. All patients included in this meta-analysis presented with an unruptured AAA and underwent elective repair.

Outcomes are reported as ORs with a 95% CI and a p-value of ≤ 0.05 was set as the threshold for statistical significance. An OR of >1 denotes that the outcome under analysis is more common following EVAR. An OR of <1, on the other hand, denotes that the outcome under analysis is more common among the OSR cohort. An OR of 1 indicates that the outcome is equally likely to occur in both treatment groups. With RCTs, all outcomes are reported on an intention-to-treat basis.

Perioperative and long-term all-cause mortality

**Figure 2:**
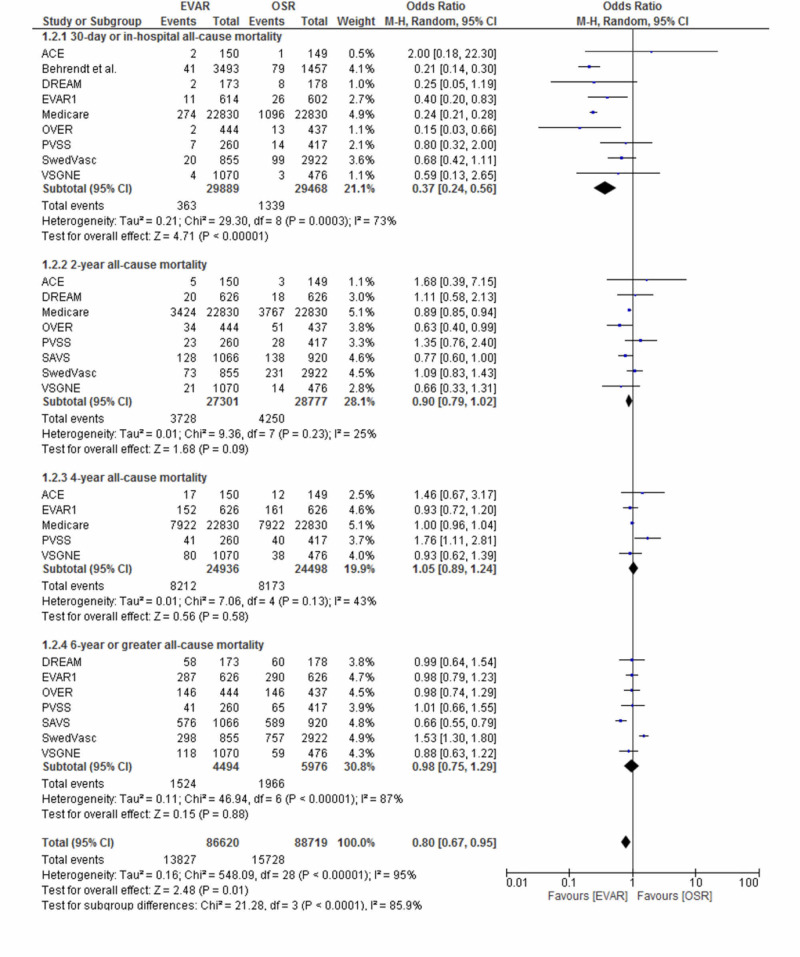
A forest plot of the odds ratio (OR) for perioperative mortality, two-year all-cause mortality, four-year all-cause mortality, and six-year or greater all-cause mortality following endovascular repair (EVAR) and open surgical repair (OSR) The estimate of the odds ratio (OR) for each clinical trial corresponds to the middle of the squares (on the right side of the figure), and the horizontal line corresponds to the confidence interval (CI). The black diamond represents the sum of the statistics and the overall OR (i.e. when all the results are pooled together). A test of the heterogeneity between the trials (I2), p-value, and total events is presented for each outcome too. ACE: Acarbose Cardiovascular Evaluation; DREAM: Diabetes Reduction Assessment With Ramipril and Rosiglitazone Medication; PVSS: Paracor Ventricular Support System; OVER: Open versus Endovascular Repair; VSGNE: Vascular Study Group of New England; EVAR1: EVAR trial 1

With the inclusion of all the clinical trials that had reported data on all-cause mortality, perioperative mortality rates, and all-cause mortality rates at two years, four years, and six years post-procedure and beyond are presented in Figure 2. Depending on the clinical trial, perioperative mortality was defined as death from any-cause 30-day post-procedure (30-day mortality) or death within the same hospital admission (in-hospital mortality).

A clear early survival advantage was seen following EVAR; a statistically significant difference, in favour of EVAR, was observed in terms of perioperative mortality (1.2% vs 4.5%; OR 0.37; 95% CI: 0.24-0.56; I2=73%; P < 0.00001). However, the early survival advantage did not persist over the course of the follow-up period. Beyond the initial perioperative period, there were no statistically significant differences in all-cause mortality rates between EVAR and OSR. All-cause mortality rates at two years (13.8% vs 15%; OR 0.90; 95% CI: 0.79-1.02; P = 0.09), four years (32.9% vs 33.4%; OR 1.05; 95% CI: 0.89- 1.24; P = 0.58), and six years post-procedure and beyond (33% vs 32.9%; OR 0.98; 95% CI: 0.75-1.29; P = 0.88) were comparable between the two treatment groups.

Aneurysm-related mortality, rupture, and reintervention 

**Figure 3:**
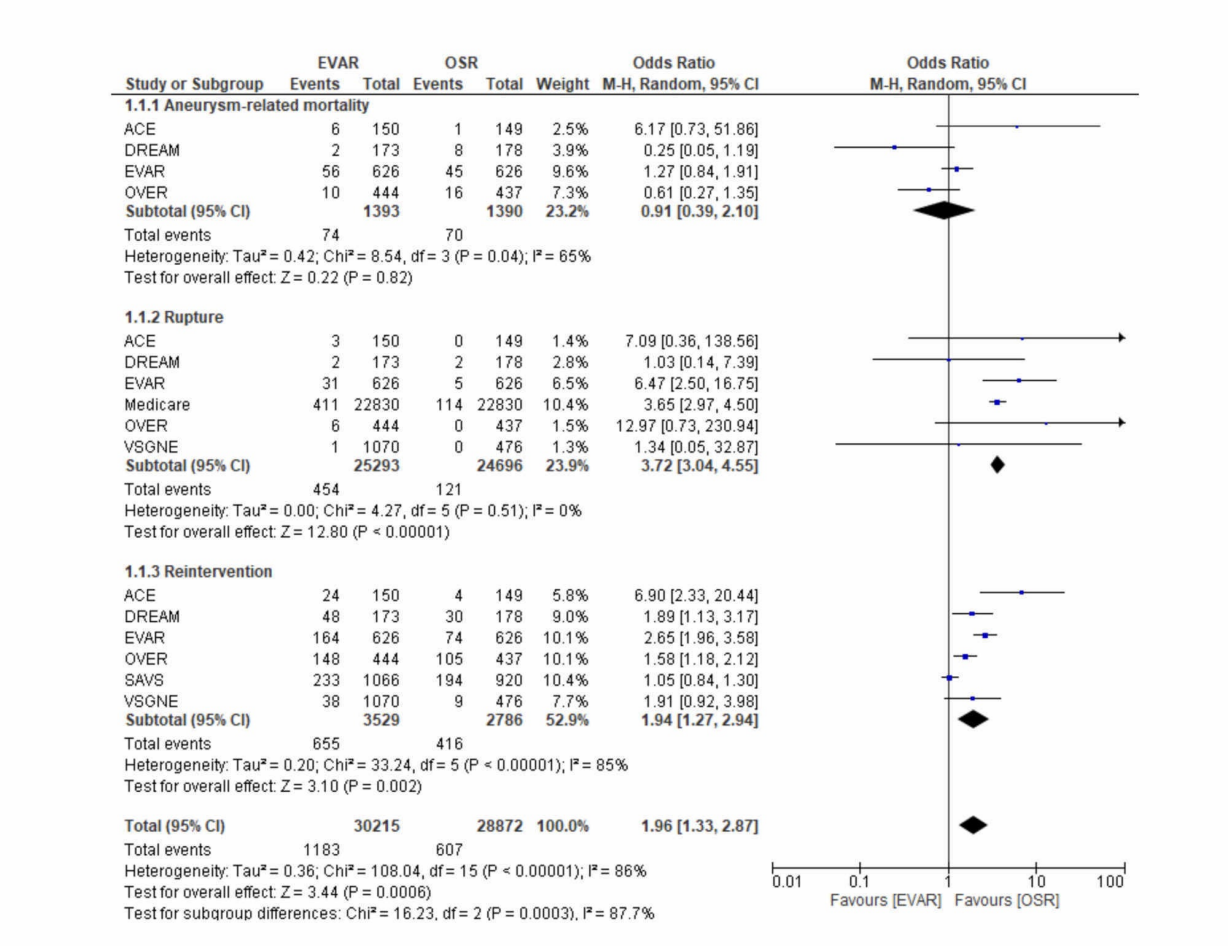
A forest plot of the odds ratio (OR) for aneurysm-related mortality, the risk of rupture, and the rate of reintervention following endovascular repair (EVAR) and open surgical repair (OSR) The estimate of the odds ratio (OR) for each clinical trial corresponds to the middle of the squares (on the right side of the figure), and the horizontal line corresponds to the confidence interval (CI). The black diamond represents the sum of the statistics and the overall OR (i.e. when all the results are pooled together). A test of the heterogeneity between the trials (I2), p-value, and total events is presented for each outcome too. ACE: Acarbose Cardiovascular Evaluation; DREAM: Diabetes Reduction Assessment With Ramipril and Rosiglitazone Medication; PVSS: Paracor Ventricular Support System; OVER: Open versus Endovascular Repair; VSGNE: Vascular Study Group of New England; EVAR1: EVAR trial 1

With the inclusion of all the clinical trials that had published results on these outcomes, data on aneurysm-related mortality, the risk of rupture, and the rate of rupture following both EVAR and OSR are presented in Figure 3.

No statistically significant differences were seen between the two treatment modalities in aneurysm-related mortality (OR 0.91, 95% CI: 0.39 to 2.10; I2 = 65%; P = 0.82). However, a significantly higher proportion of patients who underwent EVAR suffered aneurysmal sac rupture (1.8% vs 0.4%; OR 3.72, 95% CI: 3.04 to 4.55; I2=0%; P < 0.00001) and required a secondary intervention post-procedure (OR 1.94, 1.27 to 2.94; I2=85%; P = 0.002). Differences in the risk of rupture and the need for postoperative reintervention between the two treatment modalities, all in favour of OSR, remained statistically significant even after the exclusion of non-randomised trials from analysis (Appendix 1).

Postoperative complications

**Figure 4:**
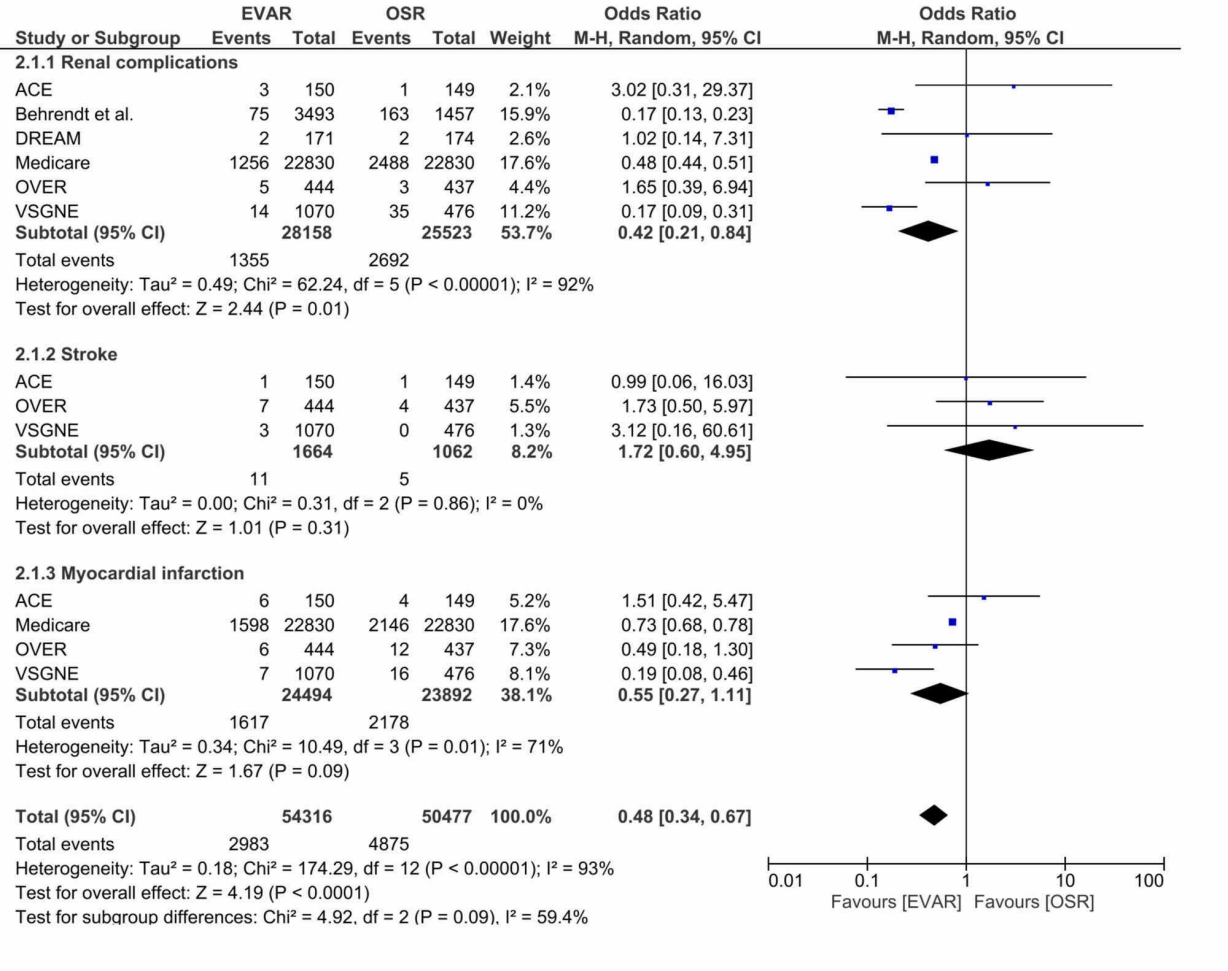
A forest plot of the odds ratio (OR) for renal complications, myocardial infarction, and stroke following EVAR and OSR. The estimate of the odds ratio (OR) for each clinical trial corresponds to the middle of the squares (on the right side of the figure), and the horizontal line corresponds to the confidence interval (CI). The black diamond represents the sum of the statistics and the overall OR (i.e. when all the results are pooled together). A test of heterogeneity between the trials (I2), p-value, and total events are presented for each outcome too. ACE: Acarbose Cardiovascular Evaluation; DREAM: Diabetes Reduction Assessment With Ramipril and Rosiglitazone Medication; PVSS: Paracor Ventricular Support System; OVER: Open versus Endovascular Repair; VSGNE: Vascular Study Group of New England; EVAR1: EVAR trial 1

Renal complications, myocardial infarction (MI), and stroke were commonly reported as postoperative complications following both treatment modalities and were subsequently selected for further comparative analysis. With the inclusion of all the clinical trials that had reported results on these outcomes, data on postoperative renal complications, myocardial infarction, and stroke following both EVAR and OSR are summarised in Figure 4.

Despite being statistically insignificant, postoperative stroke was slightly higher in the EVAR cohort as compared to OSR (0.66% vs 0.47%; P = 0.31). On the other hand, the overall rate of postoperative MI was higher following OSR, but this finding was found not to be statistically significant (9.1% vs 6.6%; P = 0.09). Although the present meta-analysis identified no statistically significant differences in the rates of postoperative MI or stroke, there was a statistically significant higher rate of postoperative renal complications among the OSR arm as compared to EVAR (10.5% vs 4.5%; I2= 92%; P = 0.01).

## Discussion

AAA represents a growing healthcare concern and is a leading cause of global mortality and morbidity. Aside from the associated increases in mortality and morbidity, the devastating personal, social, and economic consequences have all been well-established. An increasing elderly population, amongst other demographical trends, ensures the slow but steady rise in the incidence of the disease [4]. The increasing prevalence across the globe has prompted the need for a meta-analysis to enrich the existing literature and identify any gaps in the current clinical management of this disease. The present review includes clinical trials with follow-up periods of up to 12 years and provides an aggregate analysis of outcomes following both treatment modalities from the currently available body of evidence.

The inclusion of RCTs and non-randomised trials

Since RCTs are known to have limited generalisability, as patients have to eligible for both procedures in order to undergo randomisation, non-randomised comparative trials were included to determine whether the results from RCTs are likely to reflect outcomes in real-world clinical practice. A growing body of literature suggests that patients who are anatomically suitable to undergo EVAR in all randomised trials would also experience favourable outcomes following OSR. Patients deemed suitable for both procedures are also thought to have less complex aneurysms as compared to the overall population of AAA patients [5]. The limited patient heterogeneity and the superior baseline characteristics of patients may thus restrict the generalisability of RCTs.

On the other hand, the major identifiable pitfall of non-randomised trials lies within their inherent design, carrying a high risk of selection and confounding bias. Although propensity-based matching in non-randomised trials may, to a certain extent, minimise the effects of covariates, it does not remove all traces of confounding elements from the investigation [23]. Furthermore, another source of bias would be the possible link between the patient’s estimated short- and long-term risks of death, on one hand, and the decision of the vascular surgeon to advocate OSR or EVAR, on the other hand. In RCTs, this is minimised through randomisation.

The inclusion of both RCTs and non-randomised trials in the present meta-analysis thus enhances the generalisability as well as the external validity of the results and minimises any potential sources of bias. Utilising a broad inclusion criterion, the present review allows for a better understanding of the two treatment options, resulting in a more valid comparison.

Baseline characteristics 

Although attempts, to varying extents, were made in all clinical trials to ensure balanced baseline characteristics and an even distribution of risk factors across the two treatment groups, the two groups may still not be fully identical. An even distribution of risk factors across the two treatment groups does not necessarily account for the degree of association of different risk factors with the outcomes of interest (e.g. mortality). However, despite the two groups not being identical in their baseline characteristics within each clinical trial, the two groups did not differ significantly. A reasonable comparison can still be made and potential links between specific risk factors and poorer outcomes can be identified. No two patients are identical in clinical practice.

With a mean age of 74 years, the age of the patients included in this meta-analysis could be considered generalisable. The incidence of AAA increases with age, with the disease being most prevalent among those aged 65-74 and rare in young patients [24].

Perioperative mortality

An increase in mortality during and immediately following surgery represents a ‘worst-case’ scenario and is clearly a major influencing factor in terms of deciding on a surgical method. A clear survival advantage was offered by EVAR in respect to perioperative mortality (1.2% vs 4.5%; OR 0.37, 0.24 to 0.56; I2= 73%; P < 0.00001).

With the exception of the ACE (Acarbose Cardiovascular Evaluation) trial [5], all clinical trials reported a statistically significant lower perioperative mortality rate following EVAR. The ACE trial, on the other hand, reported a higher 30-day mortality rate in the EVAR arm (1.3% vs 0.6%; P > 0.05). However, having failed to recruit 50% of the target number of patients, the potential findings of this clinical trial are heavily limited by its statistical power and this finding may simply be due to a type II error. Since the OSR cohort within the ACE trial had a significantly higher Society for Vascular Surgery/American Association for Vascular Surgery (SVS/AAVS) grading score (P < 0.01), it is also possible that patients allocated to EVAR may have received less preoperative evaluations and subsequently poorer perioperative care than those receiving OSR. This may have contributed to the enhanced 30-day mortality rate in the OSR cohort. Furthermore, since there was a higher cross-over rate in the OSR arm in the trial (11.4% vs 2.7%; P < 0.01), both intention-to-treat and per-protocol analyses should have been performed for all outcome measures to obtain a well-adjusted evaluation and a balanced reflection of interventional outcomes in both groups. Despite these deficiencies within the ACE trial producing somewhat aberrant and incongruent perioperative mortality rates, this meta-analysis still demonstrates a statistically significant higher perioperative mortality rate following OSR when the results of all trials are pooled together (4.5% vs 1.2%; P < 0.00001).

Despite the apparent, clear-cut implication of this result, it is worth noting that there was a 73% heterogeneity identified (I2 = 73%). In this case, this seemingly high heterogeneity is partly owing to differences in how “perioperative mortality” was defined by the clinical trials. The DREAM (Diabetes Reduction Assessment With Ramipril and Rosiglitazone Medication) [9], Medicare [14], PVSS (Paracor Ventricular Support System) [17], and OVER (Open versus Endovascular Repair) [15] trials published a combination of 30-day and in-hospital mortality, the ACE trial [5] and the VSGNE (Vascular Study Group of New England) trial [20] reported 30-day mortality only, Behrendt et al. [6] reported in-hospital mortality only, and EVAR1 (EVAR trial 1) published a combination of 30-day and in-hospital mortality separately [11,13]. This would go some way in explaining this apparent inconsistency in the results.

Postoperative complications

Although the present meta-analysis identified no statistically significant differences in postoperative stroke or myocardial infarction following both treatments, there was a statistically significant higher rate of postoperative renal complications following OSR. Renal complications and myocardial infarction, commonly presenting perioperatively, may have contributed to the higher perioperative mortality rate seen in the OSR cohort. This is in agreement with recent studies that demonstrated postoperative renal complications and MI to independently predict perioperative mortality following successful AAA repair [25].

Although statistically insignificant, the higher postoperative rate of myocardial infarction following OSR can be reduced in the future by the administration of an intravenous bolus of heparin prior to aortic clamping. Heparin, although found not to significantly influence the risk of operative bleeding or thromboembolic complications, was found to reduce the risk of perioperative myocardial infarction from 5.7% to 1.4% [26]. Furthermore, the identification of any pre-existing cardiac abnormalities prior to surgery was also found to substantially improve survival rates and minimise any adverse cardiac events following repair. Unlike myocardial infarction, postoperative stroke was slightly higher following EVAR. Although rare and statistically insignificant, the higher incidence of stroke following EVAR, possibly associated with embolisation due to catheter guiding, can be minimised in the future by the introduction of smaller-diameter catheters and embolic-protection stent-grafts. Further analysis of long-term mortality by listed causes, as reported by a subset of clinical trials, demonstrates no statistically significant differences between OSR and EVAR in terms of postoperative CVD-related or stroke-related mortality beyond the initial perioperative period. This finding is not overly surprising, as patients who manage to survive the early perioperative period usually return to their baseline risk, irrespective of the surgical procedure undertaken.

The statistically significant higher rate of renal complications following OSR may be explained by the invasive nature of the procedure. Aortic cross-clamping during OSR is directly associated with a reduction in the renal blood flow secondary to an increase in the renal vascular resistance, subsequently reducing the glomerular filtration rate [27]. The deterioration in the GFR may persist for up to six months post-procedure. However, data on renal complications should be perused with caution. Three out of six clinical trials reporting data on postoperative renal function did not specifically define ‘renal complications’ [5,7,20]. Postoperative renal complications can range from the reversible acute kidney injury to stage 5 renal failure requiring dialysis; clearly defining renal complications in future trials would be of critical significance when balancing out the risks and complications of OSR against EVAR.

Rupture and reintervention

Given that the main aim of the surgical intervention is to prevent rupture, secondary sac rupture following either treatment option is clearly a rather unfavourable outcome that necessitates a secondary intervention. A statistically significant higher rate of rupture and reintervention was seen in the EVAR arm. These findings pose a challenge to the long-term durability of EVAR, as repair in the setting of rupture is often associated with a high mortality rate of up to 80% [28].

Given the significance of rupture, it would be prudent to explore the reasons underlying post-EVAR rupture. A possible reason includes the stent-graft devices that were used. One device common to most clinical trials was the AneurX device. The Food and Drug Administration (FDA) released a notification in 2003 recommending that this device should no longer be used in clinical settings [29]. This is natural, considering that as time moves on, technology ought to improve. Given the nature of the studies with their long follow-up periods, the results would, of course, reflect older stent-graft technology. With continuous advances in stent-graft design, it is expected that the long-term durability of EVAR would improve in the future.

Yet another possible reason behind this may be poor patient adherence to long-term postoperative surveillance. Following EVAR, ongoing surveillance is required to assess the integrity of the stent-graft and this naturally calls into question the willingness of patients to adhere to this. In the OVER trial, 50% of ruptures occurred in patients who were non-adherent to follow-up appointments [15-16]. Implantable pressure-sensing stent-graft devices can be used in the future to minimise the attendant risk of rupture following EVAR.

Although all clinical trials reporting reintervention data demonstrated a statistically significant higher rate in the EVAR arm, the post-operative reintervention rates ranged from 3.6% in the VSGNE trial [20] to 33.3% in the OVER trial [16]. Given that the likelihood of secondary sac rupture following EVAR increases with time, the differences in reintervention rates may simply be due to differences in the follow-up periods. The analysis of reintervention in the VSGNE trial was limited to one-year post-procedure, which may explain the low reintervention rate reported as compared to the OVER trial, which had a mean follow-up period of 5.2 years.

Long-term all-cause mortality

While EVAR clearly confers an early survival benefit over OSR (1.2% vs 4.5%; P< 0.00001), this early advantage did not persist beyond two years post-procedure and, therefore, does not necessarily translate into a long-term survival benefit. Beyond the initial perioperative period, no continuing survival benefit existed over the course of follow-up and the rates of all-cause mortality were comparable between the two groups at two years (13.8% vs 15%; OR 0.90; P = 0.09), four years (32.9% vs 33.4%; OR 1.05; 95% CI: 0.89-1.24; P = 0.58), and six years and beyond (33% vs 32.9%; OR 0.98; 95% CI: 0.75-1.29; P = 0.88).

The EVAR1 trial, the largest RCT included in the present meta-analysis with the longest mean follow-up period demonstrated a statistically significant higher rate of all-cause mortality at eight years post-procedure following EVAR (53% vs 46%; P = 0.048), mainly attributable to secondary sac rupture [13]. In addition to the higher risk of rupture and the need for reintervention, the higher long-term all-cause mortality beyond the initial perioperative period calls into question the long-term efficacy of EVAR in comparison to OSR. Ultimately, longer follow-up data must be presented before any definitive conclusions can be established for this potentially revolutionary technique.

The EVAR1, DREAM, OVER and ACE trials, the only RCTs included in the present meta-analysis, recruited patients from 1999-2003, 2000-2003, 2002-2007, and 2003-2008, respectively. The overall perioperative mortality rates were 3% in the EVAR1 trial [12], 2.8% in the DREAM trial [9], 1.7% in the OVER trial [15-16], and 1% in the ACE trial [5]. The two-year all-cause mortality rates were 10.8% in the DREAM trial [9], 9.6% in the OVER trial [15-16], and 2.7% in the ACE trial [5]. It is clearly evident that the later in time the trial was conducted, the lower the overall mortality rates. Since EVAR was a relatively novel surgical technique during the time these clinical trials were undertaken, there was undoubtedly a learning curve for vascular surgeons performing the surgical procedure. The relative operative inexperience, coupled with the use of early-generation stent-grafts that were more likely to cause complications, may have augmented the mortality rates and reflected poorly on the frequency of procedural complications following EVAR. This is supported by findings from recent studies demonstrating lower mortality rates and postoperative complications in higher-referral hospitals [30]. In the future, this learning curve can be minimised by localising vascular procedures in specified centres, thus ensuring adequate hands-on experience for surgeons.

The clear survival benefit conferred by EVAR is concealed by the long-term risk of rupture, reintervention, requisite hospitalisation, and the ‘catch-up’ long-term mortality. The main question at hand is whether the initial perioperative advantage afforded by EVAR is sufficient to justify the additional costs of ongoing surveillance and the higher risk of rupture and subsequent reintervention, including the conversion to OSR. Although the statistically significant difference in perioperative mortality advocates EVAR to be named the gold-standard therapy for AAAs, this statement is not entirely warranted when the risk of rupture and long-term mortality are taken into consideration. It is for this reason that one may not, as of now, be able to champion EVAR over OSR without further clinical evidence on its long-term durability, especially with increased operator experience and the emergence of new stent-graft devices. The risk of reintervention, as well as the expenses arising from these additional procedures, may offset the early survival advantage afforded by EVAR.

Conflict of interest

Of note, a subset of authors in all four RCTs included in the present meta-analysis have declared financial disclosures due to affiliation with stent-graft device companies (e.g. W.L. Gore & Associates, Inc., Newark, Delaware; and Medtronic, Fridley, Minnesota) [5,9,12,15]. Conflicts of interest undoubtedly question the neutrality of the reported data and may have had a significant influence on the results by means of reporting and publication bias. Furthermore, this inherent potential of industrial influence may have inadvertently led to conditions that were conducive to the relatively successful outcome in patients who underwent EVAR. Whilst the significant costs and logistic challenges associated with conducting large trials on relatively novel surgical procedures are recognised, and while acknowledging the potential industrial influence in this endeavour, there should be a conscious effort by vascular surgeons to perform large, well-designed clinical trials without any financial support from medical device companies. Any potential sources of industrial bias can be minimised and credibility enhanced when comparing EVAR against OSR in the management of unruptured AAAs.

## Conclusions

In conclusion, the present meta-analysis demonstrates the superiority of EVAR over OSR in terms of perioperative mortality rates. However, beyond the initial perioperative period, no survival benefit was seen over the course of the follow-up period and the early survival advantage was lost after two years post-procedure. Although the present meta-analysis identified no statistically significant differences in postoperative myocardial infarction, stroke, or aneurysm-related mortality, there was a statistically significant higher rate of renal complications following OSR and a significantly higher rate of rupture and reintervention following EVAR. In light of these findings, it would be reasonable to conclude that EVAR is as efficacious as OSR in the treatment of unruptured AAAs, but it would be invalid to claim it as superior without further clinical trials with long follow-up periods. With the current trend in healthcare systems that revolves around active patient involvement in the decision-making process, the ultimate decision regarding the type of surgical procedure should be individualised to the patient and determined on a case-by-case basis. The multidisciplinary team consisting of vascular surgeons, interventional radiologists, and cardiologists should take all the available clinical evidence, interindividual variation, and patients’ preferences and pre-existing comorbidities into account when deciding on a treatment plan aimed at the optimal restoration of the patient’s health and well-being.

## Figures and Tables

**Figure 5:**
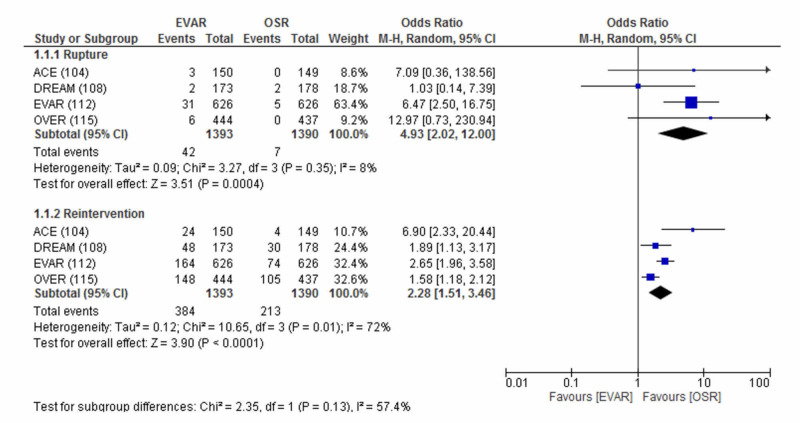
Rupture and reintervention following both EVAR and OSR with the exclusion of non-randomised trials Only RCTs are analysed in the figure. OSR: open surgical repair; EVAR: endovascular repair; RCT: randomised controlled trial; ACE: Acarbose Cardiovascular Evaluation; DREAM: Diabetes Reduction Assessment With Ramipril and Rosiglitazone Medication; OVER: Open versus Endovascular Repair

## Appendices

Appendix 1 
